# Intraoperative high-field magnetic resonance imaging combined with functional neuronavigation in resection of low-grade temporal lobe tumors

**DOI:** 10.1186/s12957-015-0690-7

**Published:** 2015-09-26

**Authors:** Shao-cong Bai, Bai-nan Xu, Shi-hui Wei, Jie-feng Geng, Dong-dong Wu, Xin-guang Yu, Xiao-lei Chen

**Affiliations:** Department of Neurosurgery, PLA General Hospital, 28 Fuxing Road, Haidian District, Beijing, 100853 China; Department of Ophthalmology, PLA General Hospital, 28 Fuxing Road, Haidian District, Beijing, 100853 China

**Keywords:** Low-grade glioma, Lesional temporal lobe epilepsy, Intraoperative magnetic resonance imaging, Neuronavigation, Seizure outcome, Return to work

## Abstract

**Background:**

The aim of this study is to investigate the role of intraoperative MR imaging in temporal lobe low-grade glioma (LGG) surgery and to report the surgical outcome in our series with regard to seizures, neurological defects, and quality of life.

**Methods:**

Patients with temporal lobe contrast-nonenhancing gliomas who presented with seizures in the course of their disease were enrolled in our prospective study. We non-randomly assigned patients to undergo intraoperative magnetic resonance imaging (iMRI)-guided surgery or conventional surgery. Extent of resection (EOR) and surgical outcomes were compared between the two groups.

**Results:**

Forty-one patients were allocated in the iMRI group, and 14 were in the conventional group. Comparable EOR was achieved for the two groups (*p* = 0.634) although preoperative tumor volumes were significantly larger for the iMRI group. Seizure outcome tended to be better for the iMRI group (Engel class I achieved for 89.7 % (35/39) vs 75 % (9/12)) although this difference was not statistically different. Newly developed neurological deficits were observed in four patients (10.3 %) and two patients (16.7 %), respectively (*p* = 0.928). Free of seizures and neurological morbidity led to a return-to-work or return-to-school rate of 84.6 % (33/39) vs 75 % (9/12), respectively (*p* = 0.741).

**Conclusions:**

Our study provided evidence that iMRI was a safe and useful tool in temporal lobe LGG surgery. Optimal extent of resection contributed to favorable seizure outcome in our series with low morbidity rate, which led to a high return-to-work rate.

## Background

Seizures are the most frequent and often the only manifestation in patients with brain tumors of glial origin, and medical treatment appears to be less effective for seizure control because of incomplete understanding of underlying pathophysiological mechanisms. Particularly, patients with slow-growing low-grade tumors (low-grade gliomas (LGGs) and glioneuronal tumors) in cortical areas of the temporal lobe are more frequently associated with seizures than high-grade tumors [1–3]. Since patients harboring low-grade tumors tend to live several years after diagnosis [4, 5], the impact of epilepsy on disease burden is high. Thus, epilepsy control is one crucial factor to improve patients’ quality of life, to free them from disease burden, and to help retain their functional independence and social participation.

It is generally believed that successful surgical resection of these tumors has a significant effect not only on the rate of overall survival (OS) and tumor progression [6–8] but also on seizure outcome. Extent of resection (EOR) has been suggested to be a strong predictor of postoperative seizure freedom [2, 9, 10]. However, LGGs are infiltrating tumors that do not have distinct borders, which cannot be easily differentiated from periphral normal brain tissue with the naked eye. Intraoperative MR imaging (iMRI) provides information about EOR and allows update of immediate data sets for neuronavigation to compensate for anatomic changes that occur as a result of surgical retraction, gravity, and loss of cerebrospinal fluid [11, 12]. Combined with neuronavigation with tractography and functional data, information about the white matter tracts and eloquent areas and their relationships with tumors can also be obtained preoperatively for evaluation and surgical design and updated during surgery. These intraoperative imaging techniques have been developed to maximize EOR while minimized postoperative neurological deficits [13–16], however, still need to be further tempered by surgical judgement.

Few studies have demonstrated the effects of iMRI on surgery of temporal lobe lesional epilepsy patients [17]. In this prospective study, we sought to investigate the value of high-field iMRI combined with integrated neuronavigation in temporal lobe LGGs resections for seizure outcome, postoperative morbidity, and rate of return to normal life and compared these findings with those of a control cohort without iMRI guidance. Initial, intraoperative, and immediate postoperative tumor volumes were measured quantitatively.

## Methods

### Study design and patients

Patients with suspected temporal lobe LGG showing no contrast enhancement on T1-weighted MRI who presented with seizures were eligible. After giving their informed consent, these cases were discussed in the morning meeting in our department, and then the patients were allocated by the chief resident to undergo either iMRI-guided or conventional microneurosurgical tumor resection (include all standard neurosurgical instruments and neuronavigation system) according to the risk and the difficulty of the operation. Only patients with histopathological examination confirmed temporal lobe grade II tumors (except gemistocytic astrocytoma) were enrolled. Functional MRI and fiber tracking were performed for both groups of patients to localize motor and speech areas and to reconstruct white matter tracts if the lesion was in the vicinity of these eloquent regions. Postoperative treatment was done according to clinical guidelines and dependent on preferences of the patient. Follow-up evaluation including neurological examination and MRI were obtained at regular intervals.

### Ethics statement

The local Ethic Committee of Chinese PLA General Hospital approved intraoperative MRI, and signed informed consent was provided by all patients or family members. We had obtained consent to publish from the participants to report their data in any form.

### MRI and data processing

Both conventional MRI and diffusion tensor imaging (DTI) were performed on a 1.5-T scanner (Siemens Espree, Erlangen, Germany). Our imaging protocol started with a T1-weighted 3D magnetization-prepared rapid-acquisition gradient-echo (MPRAGE) sequence (echo time (TE) 3.02 ms, repetition time (TR) 1650 ms, matrix size of 256 × 256, field of view (FOV) 250 mm, slice thickness 1 mm) and followed by several sequences applied in the transverse plane, which are T2-weighted sequence (TE 93 ms, TR 5500 ms, matrix size of 512 × 512, FOV 230 mm, slice thickness 3 mm); T2 fluid-attenuated inversion recovery sequence (FLAIR; TE 84 ms, TR 9000 ms, matrix size of 256 × 256, FOV 230 mm, slice thickness 3 mm); and DTI echo planar (TE 147 ms, TR 9400 ms, matrix size of 128 × 128, FOV 251 mm, slice thickness 3 mm, bandwidth 1502 Hz per pixel, diffusion-encoding gradients in 12 directions using *b* values of 0 and 1000 s/mm^2^ and voxel size 1.9 × 1.9 × 3 mm). We used 40 slices, no intersection gap, 40 continuous free interval collection slices, and five time repetitions. The same scanning protocol was used for both preoperative and intraoperative MRI.

### Fiber tracking

For reconstruction and visualization of the fiber tracts, we used the “fiber tracking” module of the navigation planning software iPlan 2.6 (BrainLab, Feldkirchen, Germany). Before tractography could be started, scans were transferred to a navigation planning workstation in DICOM format, and format conversion of the data and fusion of different sets of images were needed. The fractional anisotropy (FA) threshold was adjusted to 0.15 and the minimum fiber length to 40 mm before fiber tracking was initiated. Tract seeding was performed by defining a rectangular region of interest (ROI) in the co-registered standard T1 anatomical datasets and the color-encoded fractional anisotropy map. For reconstruction of the optic radiation, one region of interest (ROI) was placed on the lateral geniculate body identified by selecting the axial slice at the level of the transition from the posterior limb of the internal capsule to the cerebral peduncle on the resection side and a second ROI was placed at the level of the lower lip (anterior bundle) or middle and upper lip (central and posterior bundle) of the visual occipital cortex (calcarine cortex) on the same side. The arcuate fasciculus tractography was performed using approaches with two ROI, as reported by Catani et al [18]. For tractography of the pyramidal tract, we used a two-ROI approach described by Nimsky and colleagues [19, 20]. After selecting the appropriate fiber bundle, erasing false-positive tracts, a 3D object was generated automatically by wrapping neighboring fibers with a 5-mm hull. The closing lines around all fibers from all slices together resulted in the 3D object.

### BOLD imaging

For the determination of eloquent cortex, we implemented the “blood oxygenation level dependent (BOLD) mapping” module. Preoperatively, patients had to perform specific tasks including number-counting task and picture-naming task for language-eloquent cortex localization. Broca’s area was activated in the posterior section of the inferior frontal gyrus of the dominant hemisphere and Wernicke’s area in the posterior section of the superior temporal gyrus. Repeated hand and foot movement task was used activate the hand and foot cortex in the pre-central gyrus.

We used the object creation module of the iPlan 2.6 for tumor segmentation. Tumor volume was determined based on hyperintense region on T2 FLAIR because the majority of low-grade gliomas have optimal visibility in this sequence and peritumoral edema could be distinguished from the lesion. Segmentation of the tumor was performed on a slice-by-slice basis in anatomic data. After all the slices containing the lesion were outlined, a 3D object was formed.

### Microscope-based neuronavigation

Preoperative 3D T1-weighted sequence was used for intraoperative anatomic neuronavigation. Optic radiation, cortical speech areas, and other functional data, if needed according to tumor location and tumor volume, were integrated into the anatomical imaging data. After accurate registration, the neuronavigation microscope (Pentero, Carl Zeiss, Oberkochen, Germany) was connected. The functional data were overlaid in the neurosurgeon’s microscope viewing field; thus, an appropriate incision could be planned accordingly. When the surgeon had the impression of having a complete resection or further removal of the lesion might endanger eloquent areas or functional white matter tracts, an intraoperative MRI was performed. If residual tumor which could be further removed was revealed, the intraoperative MRI and DTI tractography data were used to update the navigation information, in a way compensating for intraoperative brain shift caused by tumor resection, brain swelling, and cerebrospinal fluid outflowing. The initial registration file was restored so that no repeated image registration procedure is needed. Imaging would be repeated prior to closure of the craniotomy to confirm adequate resection of any lesion and to exclude any immediate complications such as hemorrhage.

**Fig. 1:**
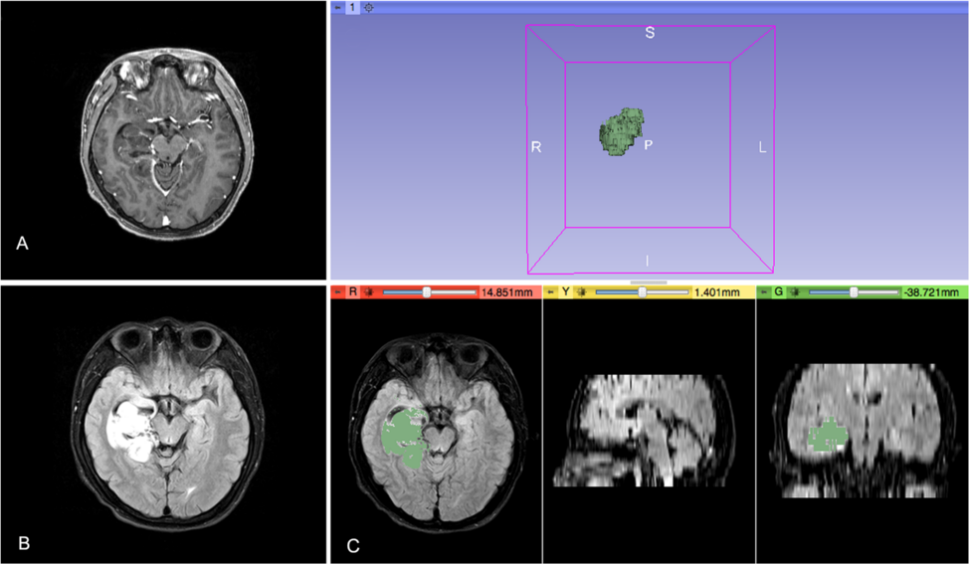
Volumetric analysis. Axial MR images obtained in a patient with a right temporal lesion infiltrating the mesial temporal lobe structures. The lesion showed an irregular shape with no enhancement on the postcontrast T1-weighted images, which was revealed an oligoastrocytoma by histopathological findings (**a**). The tumor was more visible on T2 FLAIR and was segmented manually slice by slice using the “paint effect” in the editor module of 3D slicer (**b**). Using the “make model effect,” a 3D object was built (**c**). The tumor volume automatically computed was 42.6 cm^3^

Primary endpoint was the extent of resection. To assess the extent of resection, pre- (with contrast agent within 7 days), intra-, and early postoperative 1.5T high-field MR images were collected to perform volumetric analyses. For the iMRI group, final extent of resection (EOR) was calculated based on the last intraoperative scan before closure. For the conventional group, postoperative MRI was performed within 3 days after surgery to assess the EOR. MRI scan were performed within 7 days after surgery to rule out postoperative complications such as intracranial hematoma and hydrocephalus for all patients. Volumetric analyses of the tumors and tumor residuals were undertaken by an independent and masked neuroradiologist to establish the EOR, using the editor module of 3D slicer 4.3.1 (Fig. 1). Image area of signal abnormality on T2 FLAIR sequences was segmented manually across all sections. The duration of surgery were also compared.

Secondary endpoints were neurological deficits immediately as well as 3 months and 6 months after surgery, and epilepsy outcome according to the modified Engel classification system was at 1 year [21]. A favorable seizure outcome was defined as Engel class IA–D (seizure-free or rare consciousness-sparing nondisabling seizures), whereas constant consciousness-imparing seizures were labeled as Engel classes II–IV. Postoperative neurological deficits were graded as mild (upper visual field homonymous quadrantanopia, slight aphasia, or latent mono-paresis) or severe (hemiparesis, hemianopia, or aphasia ). Deficit with no clinical improvement at the 6-month follow-up examination were considered permanent. Patients’ recent neurological and seizure outcome were gained from the follow-up examinations in our department and via telephone interviews. Return to work was obtained at 1 year after surgery.

### Statistics

Statistical analyses were performed with the software Statistical Package for the Social Sciences (version 20.0; SPSS, Inc, Chicago, Illinois). We compared binomial dichotomized data with Fisher’s exact test or *χ*^2^ test, when appropriate. Mean and median values between two groups were compared using Student’s *t* test or Wilcoxon-Mann-Whitney *U* test, when appropriate. For the iMRI cohort, correlation between potential factors and epilepsy outcome (Engel class I vs Engel classes II–IV) was analyzed using the Spearman’s rank correlation test. *P* values of less than 0.05 were considered statistically significant.

## Results

### Study population

**Fig. 2:**
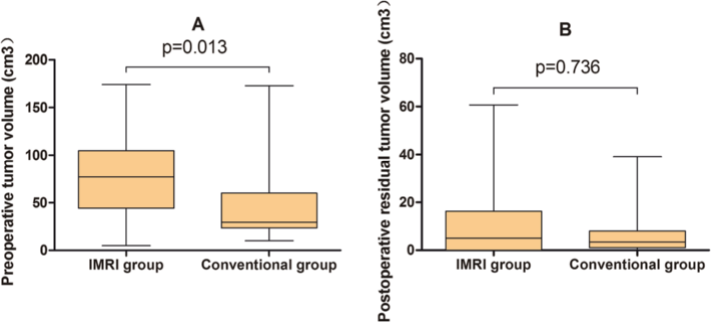
Comparison of tumor volumes between groups. **a** Preoperative tumor volumes were significantly larger for the iMRI group than the conventional group (*p* = 0.013). **b** There were no significant differences for the two groups in terms of residual tumor volumes (*p* = 0.736)

We enrolled 59 epilepsy patients who were operated on in our department between March 2009 and September 2013. Forty-six patients underwent surgery in the iMRI suite, but five patients were excluded from the study. Postoperative histopathological examination revealed three of theses patients had anaplastic astrocytoma; one patient had parenchyma inflammation. One patient with gemistocytic histology was excluded to create a more uniform study population. Thirteen patients underwent conventional surgery. Thus, data from 54 temporal lobe World Health Organization (WHO) grade II glioma patients with preoperative seizures could be analyzed (Table 1). In our series, 59.3 % of patients were male, and complex partial seizure was the predominant type. Before surgery, three patients in the iMRI cohorts were taking antiepileptic medications and one of them experienced intractable seizures. None were taking AEDs for the conventional cohorts. Median preoperative tumor volumes differed significantly between groups (77.0 cm^3^ (range 5.0–174.0) for the iMRI group and 29.5 cm^3^ (range 10.0–172.9) for the control group, *p* = 0.011; Fig. 2). Larger tumor was reflected as tumor infiltration beyond temporal lobe, with 34 patients (82.9 %) infiltrating the insular cortex in the iMRI cohorts vs 4 patients (30.8 %) in the other cohorts (*p* = 0.001). Comparison showed significant difference with respect to sub-location: 37 (90.2 %) patients in the iMRI group and 8 patients (61.5 %) for the other group showed mesial structure involvement (*p* = 0.046). There were no significant differences with regard to all other presurgical factors between the two groups. Two patients in the iMRI group and one in the other were lost to follow-up.

**Table 1:** Patients’ demographics

	Intraoperative MRI group (*n* = 41)	Conventional treatment group (*n* = 13)
Sex (male)	24 (58.5 %)	8 (61.5 %)
Mean age (range)	36.8 (16–66)	39.4 (11–50)
Site of resection (left)	15 (36.6 %)	4 (30.8 %)
Tumor infiltration		
Temporal lobe only	5 (12.2 %)	9 (69.2 %)
Insula	34 (83.0 %)	4 (30.8 %)
Frontal	24 (58.5 %)	4(30.8 %)
Sub-location		
Mesial only	23 (56.1 %)	5 (38.5 %)
Lateral only	4 (9.8 %)	5 (38.5 %)
Both	14 (34.1 %)	3 (23.1 %)
Histology		
Astrocytoma	16	3
Oligodendroglioma	10	5
Oligoastrocytoma	15	5
Type of seizures		
Complex partial	22 (53.7 %)	8 (61.5 %)
Generalized tonic-clonic	20 (48.8 %)	6 (46.2 %)
Simple partial	5 (12.2 %)	–
Absence	3 (7.3 %)	–
Seizure duration in months (range)	3 (0.25–108)	4.5 (0.25–84)
Median tumor volume in cm^3^ (range)	77.0 (5.0–174.0)	29.5 (10.0–172.9)
Median follow-up in months	32.5 (9–59)	33.3 (10–64)

### Volumetric analysis

In the iMRI group, surgery was terminated in 23 patients (56 %) after first intraoperative scan. Gross total resection (GTR) was accomplished in 7 of these patients (30 %). The other 16 patients (70 %) underwent no further resection owing to tumor infiltration of eloquent cortex or fiber bundles. The median preoperative and residual tumor volumes were 77.6 cm^3^ (range 4.9–174.0 cm^3^) and 7.9 cm^3^ (range 0.0–60.7 cm^3^), respectively. The median EOR for all 23 patients was 88.6 % (range 65.1-100 %). In 18 patients (44 %) who underwent further resection of residual tumor after first iMRI scan, GTR was achieved after additional tumor removal in 6 of the 18 patients (33 %). The median preoperative, residual tumor volumes after first iMRI and final iMRI were 68.5 cm^3^ (range 14.8–163.3 cm^3^), 17.5 cm^3^ (range 2.6–69.9 cm^3^), and 3.1 cm^3^ (range 0.0–43.6 cm^3^). The median percentage of tumor resection were increased from 71.1 % (range 53.7–95.5 %) after the first scan to 94.4 % (range 73.3–100 %) determined from the last iMRI (*p* < 0.001). In these patients, second iMRI scan revealed residual tumor resectable in two patients, so a third scan were performed before finishing surgery.

When all 41 patients were evaluated, the median EOR determined from the final iMRI was 92.5 % (range 65.1–100 %). As a result, the total number of patients undergoing GTR in the iMRI group was 13 (32 %); in 6 of them (46 %), iMRI contributed directly to this achievement.

In the conventional group, the median residual tumor volume was 3.4 cm^3^ (range 0.0–39.1 cm^3^), and median EOR was 90.7 % (range 68.7–100 %) in which GTR was achieved in 2 patients (15.4 %). No significant difference was found between the two treatment groups with regard to residual tumor volume (*p* = 0.736; Fig. 2) and EOR (*p* = 0.634).

### Surgery duration

**Fig. 3:**
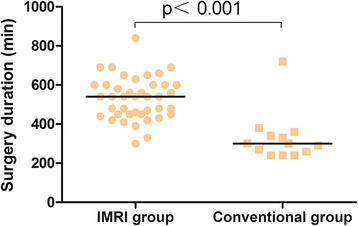
Comparison of time of surgery between groups. Surgery durations were significantly longer for the iMRI group. The one patient with exceptional longer time of surgery in the conventional group was caused by hemostasia difficulty

Duration of surgery, measured from skin incision to wound closure, was significantly longer in the iMRI group (median 540 min (range 300–840)) than it was in the conventional group (median 300 min (range 240–720)), due to further removal of residual tumor and preparing and scanning time needed in the iMRI group (*p* < 0.001; Fig. 3). Only one patient in the conventional group underwent surgery over 7 h, owing to hemostasia difficulty.

### Postoperative course

At the most recent follow-up, 35 of the 39 patients (89.7 %) in the iMRI group were stable and 4 (10.2 %) had progressed (including 2 deaths), compared with 9 of the 12 patients (75 %) who were stable in the other group and 3 (25 %) who had progressed and died (*p* = 0.413). Postoperative aggravated or new neurological deficits were observed in 15 of 51 patients (29.4 %). Twelve of 39 patients were in the iMRI group in which 8 patients returned to normal at the first follow-up visit, and 3 of 12 patients were in the conventional group (Table 2). Permanent deficits developed in 4 patients (10.3 %) in the iMRI group, compared with 2 patients (16.7 %) in the conventional group (*p* = 0.928). Intraoperative imaging had led to further tumor resection in 2 of the 4 patients with permanent neurological deficits. Complications that were directly related to the intraoperative MRI scan were not observed, particularly postoperative infection. One patient developed intracranial hemorrhage the day after surgery that required immediate surgical evacuation.

**Table 2:** Postoperative complications

	Transient	Permanent
iMRI group		
Focal motor deficit	8^a^ (20.5 %)	2^a^ (5.1 %)
Aphasia	1 (2.6 %)	1 (2.6 %)
Quadrantanopia	4^a^ (10.3 %)	2^a^ (5.1 %)
Conventional group		
Hypoesthesia	1 (8.3 %)	1 (8.3 %)
Oculomotor paralysis	1 (8.3 %)	1 (8.3 %)
Quadrantanopia	1 (8.3 %)	0

^a^One patient had both a focal motor deficit and quadrantanopia

### Seizure outcomes

**Fig. 4:**
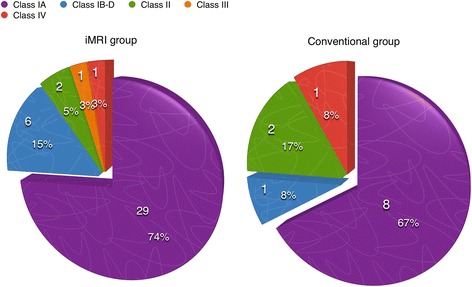
Engel classification outcome of the two groups of patients

Seizure outcomes tended to be better for patients in the iMRI group than for the conventional group, although this difference was not statistically significant (Engel class I for 89.7 % (35/39) vs 75 % (9/12), respectively; *p* = 0.413; Fig. 4). Good seizure control is reflected as a good rate of return to work: for the iMRI group, 33 patients (84.6 %) returned to work after surgery for the iMRI group, in which an Engel class I seizure outcome were achieved in 32 patients and an Engel class II in 1 patient, and 9 patients (75.0 %) returned to work for the conventional group, in which an Engel class I outcome was found in all of them (*p* = 0.741).

Spearman rank correlation analysis revealed that seizure outcome (Engel class) was related to increased EOR (*r* = −0.452, *p* = 0.004) and larger tumors (*r* = 0.391, *p* = 0.014). Significant correlation between seizure outcome and factors with regard to age and preoperative seizure history were not observed.

### Illustrative case

**Fig. 5:**
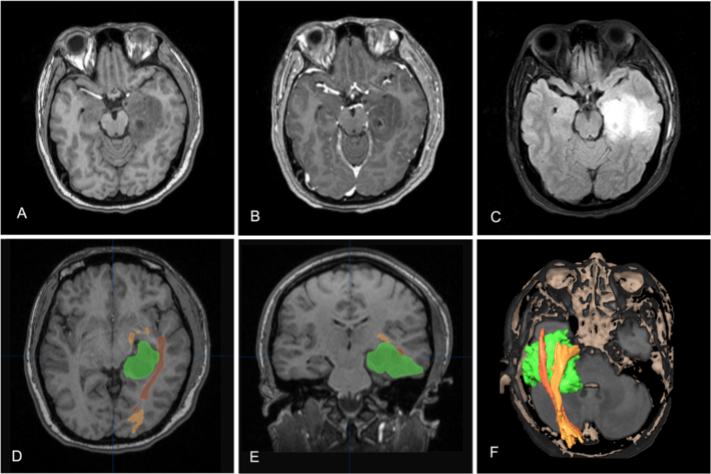
Preoperative MR imaging. Conventional MRI showed a lesion located in the left temporal lobe involving the mesial temporal structures with no contrast enhancement (**a**–**c**). The reconstructed optic radiation was rostral to the lesion, and Meyer’s loop was closely involved in the lesion (**d**, **e** *orange*, dorsal bundle; *brown*, Meyer’s loop). The 3D relationship between the lesion and the optic radiation is shown (**f**)

**Fig. 6:**
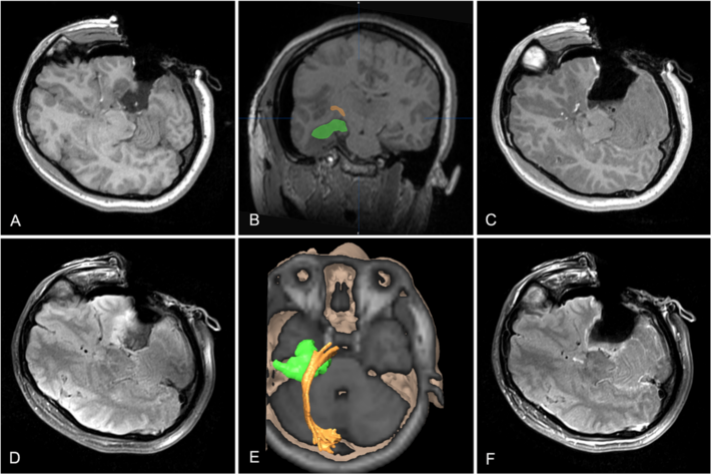
Intraoperative MR imaging. The same patient as in Fig. 1. The first iMRI scan demonstrated that the partial lesion prior to the cavity remained (**a**, **d**). After infusion of the intraoperative optic radiation tractography and the outlined residual tumor, the neuronavigation was updated (**b**, **e**). Final iMRI scan revealed that the tumor remnants had been removed (**c**, **f**)

**Fig. 7:**
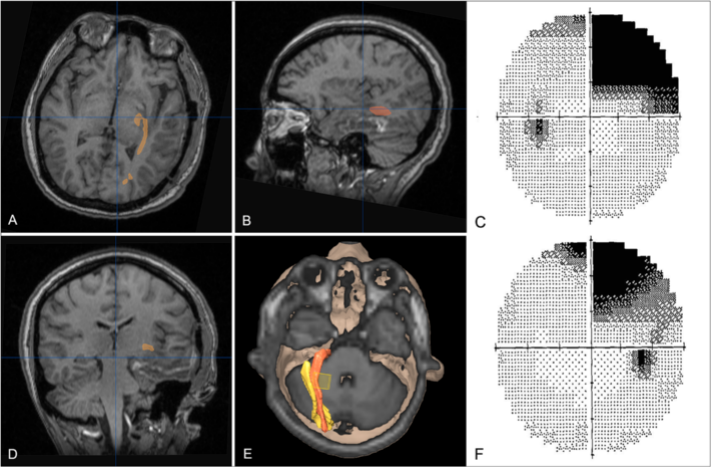
Examination 1 week after surgery. The same patient as in Figs. 1 and 2. Postoperative MRI showed that the dorsal bundle of the optic radiation was well preserved, while anterior tip of Meyer’s loop was damaged (**a**, **b**, **d**, **e**; *brown*, dorsal bundle; *yellow*, Meyer’s loop). The patient developed right superior quadrantanopia of both eyes (**c**, **f**)

A 26-year-old female had a complex partial seizure 2 weeks before surgery. Preoperative MRI revealed a nonenhanced lesion in the left temporal lobe (Fig. 5a–c), and tractography identified the optic radiation passing rostral to it and was pushed slightly upward. The minimum distance between the lesion and dorsal bundle of the optic radiation was >5 mm, while Meyer's loop was closely involved in the lesion (Fig. 5d–f). The option between a more conservative resection (lower risk to visual field) vs a gross-total resection (certain damage to Meyer’s loop but greater chance of seizure freedom and reduced risk of tumor recurrence) was discussed, and the patient chose the latter. A left inferior temporal approach was planned, and a suitable cortex incision was designed according to neuronavigation. First, iMRI detected partial tumor remnants (Fig. 6a and d), and intraoperative DTI-based dorsal bundle of the OR was reconstructed (Fig. 6b and e). After updating the neuronavigation, further resection of the lesion continued. Final intraoperative scan showed that the lesion was completely removed (Fig. 6c and f). The pathological diagnosis was World Health Organization grade II astrocytoma. According to the imaging 1 week after surgery, dorsal bundle of the OR was intact, while anterior portion of Meyer’s loop was unable to reconstruct (Fig. 7a, b, d, e). The patient was seizure free at two and a half years’ follow-up and had a right superior quadrantanopia (Fig. 7c and f).

## Discussion

Low-grade gliomas are infiltrative tumors which progressively invade the brain tissue by migrating along the subcortical white matter tracts. Contrary to the indolent characteristics claimed by classical literature, there is a constant growth pattern of these tumors before and after surgery in cases of incomplete resection [22]. Tumor progression will inevitably occur, which will lead to neurological deficits with a deteriorating of quality of life and ultimately to death. Since surgery is the first therapeutic option to consider in LGG, the impact of surgery has been studied in numerous literature, supporting the significant effect of EOR on overall survival (OS) by delaying anaplastic transformation, even in incomplete tumor removal [7, 8, 23]. However, LGG tends to have ill-defined margin which is nearly impossible to distinguish from peritumoral normal brain under the microscope with the naked eye, and EOR tend to be overestimated even by experienced neurosurgeons.

Intraoperative MRI is today an increasingly proved useful tool for operations of many brain lesions, especially for small deep seated tumors. This technique provides nearly real-time images, which allows detection of residual tumors and immediate intraoperative complications. Furthermore, it enables intraoperative update of registration compensating for brain shift caused by outflow of cerebrospinal fluid, deformation from retraction and effects of gravity [12].

### Extent of resection

For discussion of impact of iMRI on glioma surgery, the resection control was the most relevant feature which has been validated by different studies [13, 14, 24, 25]. And the advantage provided by iMRI for further reduction of tumor volume was obvious. One prospective volumetric analysis enrolled both low and high-grade glioma cases and reported that EOR of contrast-nonenhancing tumors benefit more from iMRI scan than enhancing tumors, possibly due to diffuse signal abnormality of nonenhancing gliomas on T2-weighted images. The median percentage of tumor resected in patients with nonenhancing gliomas improved from 63 to 80 %, although this was not statistically significantly due to small sample size [24]. In our series, we uniformly enrolled WHO grade II gliomas focusing on temporal lobe location. As a consequence of iMRI imaging, we identified 44 % of patients with residual tumors intraoperatively and continued surgery using the updated neuronavigation data, and EOR of these patients increased significantly from 71.1 to 94.4 %. Final iMRI scans documented gross-total resection rate of 31.7 %, in which 14.6 % of improvement benefited directly from iMRI. Compared to another study investigating impact of iMRI on lesional temporal lobe epilepsy surgery, this seems low [17]. However, WHO grade I tumors (GGs and DNTs) made up the majority of their cases, which might be less infiltrative than LGG and tumor borders might be clearer. Further, they adopted qualitative categorization of EOR instead of volumetric manual segmentation of the tumor. It is likely that tumor volume were limited in their series; thus, tumors were less involved with eloquent areas. Of note, inter-observer variances exist even for the quantitative approach, which theoretically has the advantage of higher precision [26]. Stratified analysis with regard to pathological diagnosis and tumor location using validated criteria to assess EOR are needed for comparisons of results between studies in the future.

A randomized, controlled trial was conducted aiming to assess the efficacy of iMRI guidance on extent of resection in patients with glioma [25]. They randomly allocated patients with contrast enhancing gliomas to undergo tumor resection either with iMRI guidance or with conventional microneurosurgical instruments and techniques. Their data showed significantly better extent of resection and decreased residual tumor volume in the iMRI group than in the control group, and this benefit was associated with an extended progression-free survival (PFS). However, we were unable to conduct a randomized trial due to cost-effective considerations. We would only propose an iMRI-guided surgery if preoperative imaging data had presented a large tumor with diffuse component in close relationship with functional subcortical white matter tracts because an iMRI-guided surgery was quite costly. This resulted in the larger tumor volumes in the iMRI group and highly uneven number of observations between the two groups. However, although selection bias set up a challenge for the resection in the iMRI group, comparable results were achieved with regard to final residual tumor volume and EOR, which could reflect indirectly the beneficial effect of guidance with iMRI for complete resection of LGGs compared with conventional microsurgery. Our study was not powered or designed to show whether this benefit corresponded to an increase in PFS and OS.

### Functional neuronavigation

As has been reported, BOLD MRI and DTI-based fiber tracking were noninvasive tools to localize eloquent gray matter and white matter tracts which can be integrated into a neuronavigation system. Eloquent cortex including the primary motor regions and Broca and Wernicke areas and functional subcortical connectivity including pyramidal, language, and visual tracts were successfully created and implemented in all patients in the iMRI cohorts as needed to reduce the potential risk of damage to these structures [12, 16, 20, 27, 28]. When the process of fiber tracking was done, we tended to add a 5-mm hull around the object. During surgery, the surgeon would be reminded that the manipulation was getting closer to the object if the contouring line turned from dotted to solid under the microscope. Sometimes, we had to leave some residual tumor in the brain when trying to dissect the tumor boundaries from the tracts in cases of relatively severe compression. When the surgeon had the impression of a satisfactory resection or further resection might pose a threat to functional region, an iMRI scan was performed to evaluate the EOR and update information acquired for the neuronavigation. In terms of neurological outcome, our results were more favorable than previous literature [23, 29, 30], with permanent mono-paresis occurred in two patients, upper visual field defects in two patients, mild aphasia in one patient, and complications occurred in two patients in whom iMRI led to continued resection of tumor tissue. This underlined the fact that the application of multimodal navigation combined iMRI was a safe and advantageous technique. Reviewing preoperative surgical planning of integrated anatomical and functional data of the two mono-paresis patients, we found rather severe tumor compression of the posterior limb of internal capsule, and the pyramidal tract reconstructed presented an obvious inward curve. Of note, our data showed that surgery in the iMRI operation room consumed significantly more time than it did in other rooms, not due to only continued resection but also preparation and scanning time. Fortunately, this did not resulted in wound infection in any patient. For the conventional group, two patients developed permanent postoperative defects. Preoperative MRI revealed a mesial temporal lobe lesion invading the interpeduncular fossa and prepontile cistern for the patient who had postoperative oculomotor paralysis.

### Tumor-related epilepsy

Given the fact that low-grade gliomas are slow progressing tumors which are more frequently associated with symptomatic focal seizures than high-grade gliomas, epilepsy usually represents the key clinical issue in these patients. Although surgery is facilitated by advances in preoperative and intraoperative techniques such as multimodal integrated neuronavigation, iMRI and electrical cortical and subcortical stimulations to perform a safe and maximal resection even for eloquently located LGGs, surgery cannot cure these patients. Good seizure outcome and increased quality of life in these patients with many years of survival expectancy should remain a priority. However, whether observational waiting until the occurrence of drug-resistant epilepsy or early resection irrespective of seizure control should be proposed has long been discussed [31]. From oncologic observations, convincing evidence has been provided that early surgery significantly improves PFS and OS [32]. Meanwhile, postoperative seizure outcome correlated with preoperative seizure history [9], so presumably, a timely surgery might also prove optimal for seizure control. Our study reflected a cohort routinely seen in a neuro-oncology clinic and did not focus only on patients with intractable epilepsy. Most patients in our study had sporadic seizures without long-term regular medications. As a result, duration between seizure onset and surgery was significantly shorter compared with other studies, which was considered to be one of the factors contribute to our exceptional seizure outcome [17, 30]. However, we did not observe an influence of seizure history on seizure outcome as others did [2, 9, 33], possibly attributable to the small sample size and fewer poor outcomes in our population.

Tumor-related epilepsy had unknown mechanisms, and several hypotheses for epileptogenesis including blood-brain barrier disruption, metabolic changes due to vascular disturbance, and functional connectivity alteration had been proposed [34]. Since these changes might take place not only within the tumor but also in peritumoral area or even distant from the tumor, the epileptogenic zone was not always resected with oncological surgery. Thus, conventional oncological surgery might not be sufficient to achieve a desirable seizure outcome, and consideration should be given to a more extended resection, especially in patients with tumors in or around temporal lobe because mesial temporal structures might become an independent seizure generator and may be responsible for postoperative persistent seizure activity [35]. In one meta-analysis, Englot et al. reported additional benefit with regard to seizure outcome for tailored resection including hippocampectomy plus corticectomy over gross-total lesionectomy alone [10]. However, we noticed in the literature that the association between tumor and hippocampal sclerosis (HS) is rare, with HS coupled with a tumor occurred in only 7–8 % of cases [36, 37]. Further, some studies reported similar seizure outcome between groups regarding the resection of hippocampus and amygdala [30, 37]. When trying to explain the similar results achieved in temporal lobe epilepsy surgery using different approaches with different extent of resection of temporal lobe mesial structures, Abosch et al. hypothesized that it would be enough to sever or interrupt a large enough proportion of the network between these structures, so that a seizure is unable to be built up or sustained [38]. In addition, the insular cortex was associated with seizure spread [3]. This is probably why satisfactory seizure outcome could be achieved by some studies performing lesionectomy alone or even subtotal tumor resection [39, 40] and why mesial temporal location prognosticated favorable seizure outcome in tumoral epilepsy patients [9]. In our series, excluding patients that were lost to follow-up, 82.1 % of patients in the iMRI group had tumor invasion of the insular cortex and 89.7 % of patients had tumor involvement of the mesial structures of temporal lobe, which was significantly higher than that in the conventional group (insular cortex involvement: 25.0 %; mesial structure involvement: 58.3 %). Reducing an epileptogenic mass involving these regions was more likely to include excision of the mesial temporal structures and the insular cortex and might possibly resulting in disconnection of critical seizure propagation pathways. This was considered to be another factor contributing to our favorable seizure outcome.

### Return to work

Return to work could be used as a surrogate parameter for patients’ good performance status as well as improved quality of life. It required not only a high degree of independence but also spared cognitive functions. Among patients in our iMRI cohort, 84.6 % had returned to work or school within 1 year after surgery. This rate of return to work was comparable to a previous study, which reported 90.3 % of patients returned to work 6 months after surgery in a cohort with frontal lobe LGG patients, indicating that return to previous lifestyle can be achieved for most LGG patients after surgery [41]. In our iMRI cohort, other problems including neurological defects and tumor progression prevented patients who had an Engel class I seizure outcome from returning to work in 3 patients. Three patients had not returned to work in the conventional group, all due to tumor progression. Thus for the relatively young patients with tumor-related epilepsy patients, complete seizure freedom without postoperative adverse events was the ultimate goal of each resective procedure.

### Limitation

One major limitation of the study was that it was not done in a double-blinded, randomized manner, which allowed the existence of selection bias. It also resulted in the highly uneven numbers of observations between the groups. However, even the patients with larger tumors were allocated to undergo surgery in the iMRI operation room; similar results were observed with regard to EOR, and although not statistically significant, even better results for the iMRI group with regard to seizure outcome. This is done without the compromise of patients’ postoperative neurological function, indicating the advantage of iMRI-guided surgery over conventional approach. Our finding might also be restricted by the fact that our study was done at only one center, which resulted in possible admission bias. Still, we believed that our results were valid and provided additional information for the most controversial topics in lesional temporal lobe epilepsy surgery.

## Conclusions

In conclusion, the best surgical approach to temporal lobe LGG-related epilepsy is still debated. Timely surgery before the seizure becomes medically intractable is advisable. More aggressive resection of the infiltrative temporal lobe WHO grade II tumors which frequently involving the mesial structures and even the insular cortex may contribute to a satisfactory long-term seizure outcome. iMRI is a safe and appropriate method to improve the extent of resection of LGGs. The multimodal intraoperative imaging technique led to a favorable Engel class I outcome in our study with low morbidity rate. In majority of temporal lobe LGG-related epilepsy patients, surgery will free them from seizures, and they will retain their functional independence and normal state of social life.

## Abbreviations

BOLD: blood oxygenation level dependent
DTI: diffusion tensor imaging
EOR: extent of resection
FA: fractional anisotropy
FLAIR: fluid-attenuated inversion recovery sequence
FOV: field of view
iMRI: intraoperative magnetic resonance imaging
LGGs: low-grade gliomas
MPRAGE: magnetization-prepared rapid-acquisition gradient-echo
OS: overall survival
PFS: progression-free survival
ROI: region of interest
TE: echo time
TR: repetition time
WHO: World Health Organization
